# Séroprévalence de la cysticercose et facteurs de risque associés chez un groupe de patients vus au Centre Hospitalier Régional de Référence d’Antsirabe, Madagascar

**DOI:** 10.11604/pamj.2017.28.260.10463

**Published:** 2017-11-23

**Authors:** Norosoa Julie Zafindraibe, Jeannine Ralalarinivo, Andriamiarimbola Irène Rakotoniaina, Muriel Nirina Maeder, Mala Rakoto Andrianarivelo, Bénedicte Contamin, Alain Michault, Andry Rasamindrakotroka

**Affiliations:** 1 Centre d’Infectiologie Charles Mérieux, Antananarivo, Madagascar; 2 Faculté de Médecine, Université d’Antananarivo, Madagascar; 3 Centre Hospitalier Régional de Référence d’Antsirabe, Madagascar; 4 Fondation Mérieux, Antananarivo, Madagascar; 5 Laboratoire de Bactériologie, Virologie et Hygiène Hospitalière, Groupe Hospitalier Sud Réunion, Ile de la Réunion

**Keywords:** Séroprévalence, cysticercose, Taenia solium, Western Blot, Madagascar, Seroprevalence, cysticercosis, Taenia solium, Western blot, Madagascar

## Abstract

**Introduction:**

A Madagascar, la cysticercose, maladie causée par la forme larvaire de *Taenia solium*, demeure un problème de santé publique. En 2003, la séroprévalence de la cysticercose variait entre 7% et 21% avec un taux plus élevé dans les régions centrales de l’île. Toutefois, depuis une dizaine d’année, les données épidémiologiques concernant la cysticercose humaine restent limitées. Notre étude a pour objectif de déterminer, chez les patients issus de la région de Vakinankaratra et qui sont suspects cliniquement, la séroprévalence de la cysticercose par Western blot ainsi que les facteurs de risque associés.

**Méthodes:**

Il s’agit d’une étude descriptive transversale menée sur une période de 6 mois au sein du Centre Hospitalier Régional de Référence d’Antsirabe. Tous les patients inclus dans l’étude ont répondu à un questionnaire clinique recueillant leurs caractéristiques sociodémographiques et culturelles ainsi que leurs habitudes alimentaires et leurs symptômes cliniques.

**Résultats:**

La séroprévalence de la cysticercose retrouvée dans la population d’étude était de 14,8% (35/237). Ce taux ne diffère pas significativement selon le sexe, l’âge, la consommation de viande de porc ou le mode de préparation de la viande (p > 0,05). Par contre, une différence significative (p < 0,05) a été observée chez les sujets présentant des nodules sous-cutanés ou un résultat de cysticercose antérieur positif.

**Conclusion:**

L’index élevé d’exposition à *Taenia solium* retrouvé dans notre étude justifie le renforcement des mesures de contrôle et de prévention déjà implantés dans le pays.

## Abstract (en)

**Introduction:**

In Madagascar, cysticercosis, a disease caused by Taenia Solium’s larval form, is a public health problem. In 2003, the seroprevalence of cysticercosis ranged between 7% and 21% with a higher rate in the central regions of the island. However, epidemiological data on human cysticercosis have been limited over the last dozen years. The objective of our study aimed to determine the seroprevalence of cysticercosis Western blot as well as its associated risk factors in patients from the region of Vakinankaratra with clinical suspicion.

**Methods:**

We conducted a cross-sectional, descriptive study at the Regional Referral Hospital in Antsirabe over a period of 6 months. All the patients included in the study answered a clinic questionnaire collecting their socio-demographic and cultural features as well as their dietary habits and their clinical symptoms.

**Results:**

The seroprevalence of cysticercosis in the study population was 14.8% (35/237). These rates did not differ significantly according to sex, age, pork consumption or meat preparation (p > 0.05). However, a significant difference (p < 0.05) was observed among patients with subcutaneous nodules or positive for cysticercosis.

**Conclusion:**

In our study, the high index of exposure to Taenia solium justifies the strengthening of control and prevention measures already set up in our country.

## Introduction

La cysticercose, maladie causée par la forme larvaire de *Taenia solium (T.solium)*, reste encore de nos jours un problème de santé publique dans plusieurs pays à faible revenu. Avec la taeniase, elle forme un complexe parasitaire endémique dont plusieurs foyers ont été identifiés en Amérique latine, en Asie et en Afrique [1-3]. Selon l’Organisation Mondiale de la Santé (OMS), la cysticercose représente une pathologie potentiellement éradicable mais encore négligée [4]. Cette maladie est due à différents stades de l’infection à *T. solium* incluant le porc comme hôte intermédiaire et l’homme comme hôte définitif ou intermédiaire. Les porcs acquièrent la cysticercose en ingérant les œufs de T. solium contenus dans les fèces du porteur de Tænia [5]. Tandis que chez l’homme, elle est due essentiellement à l’ingestion d’eau ou d’aliments contaminés par les œufs de *T. solium* et parfois lors de l’ingestion accidentelle d’œufs de tænia par auto-infestation [6]. Les cysticerques vont alors se développer au niveau des muscles, des yeux et du système nerveux central. Toutefois, le système nerveux central constitue chez l’homme l’un des principaux sites de migration des larves causant la neurocysticercose. Cette pathologie est responsable de plus de 30% des épilepsies acquises dans les pays endémiques avec une morbi-mortalité élevée [7]. D’autres manifestations isolées peuvent également être observées, telles que la cysticercose oculaire ou musculaire [8].

A Madagascar, les mesures d´hygiène élémentaires ne sont pas respectées. Ainsi, l’utilisation des latrines est très rare et les équipements sont bien souvent absents en zone rurale. Selon l’UNICEF, seuls 11% des ménages ont accès à des installations sanitaires adéquates et près d’un tiers de la population fait ses besoins dans la nature [9]. Les défécations humaines se retrouvent ainsi régulièrement près des terrains cultivés. L’élevage porcin est commun et représente une occupation répandue dans plusieurs régions de Madagascar. Il est d’ailleurs plus important dans les régions centrales de l’île [10]. Les porcs sont ainsi généralement élevés en liberté dans les villages où ils peuvent ingérer les fèces humaines. La coexistence des mauvaises conditions sanitaires et de l’élevage porcin en liberté jouent ainsi un rôle important dans la circulation de *T. solium* au sein de la population. Le porc, hôte intermédiaire naturel, représente donc l’élément crucial dans l’entretien du cycle de *T. solium* [11,12]. En 2003, la séroprévalence de la cysticercose est évaluée entre 7% et 21% avec un taux inférieur à 10% dans les régions côtières (Mahajanga et Toamasina) et plus élevé dans les régions centrales de l’île (Antananarivo et Fianarantsoa). Les enquêtes menées dans la région des Hautes-Terres où l’élevage familial de porc est intense, ont donné des prévalences de la cysticercose supérieures à celles menées dans les régions côtières où l’élevage de porc est précaire et où la consommation de viande de porc est généralement taboue [13]. Afin de mettre à jour les données épidémiologiques concernant la cysticercose humaine à Madagascar et de renforcer les programmes de lutte déjà menées par le Ministère de la Santé Publique, la présente étude a été menée dans le but de déterminer par Western blot la séroprévalence de la cysticercose chez les patients cliniquement suspects de cysticercose dans la région de Vakinankaratra. Les objectifs secondaires étaient de décrire les caractéristiques sociodémographiques des patients testés et d’identifier les facteurs de risque de séropositivité.

## Méthodes

### Site de l’étude

Située sur les Hautes-Terres, la région de Vakinankaratra s’étend sur une superficie de 16.599km^2^ avec une densité globale de population estimée à 95,8 habitants/km^2^. L’effectif de la population est toutefois plus important en milieu rural (77,72%) qu’en milieu urbain. Le chef-lieu, Antsirabe, se situe à 162km au sud de la capitale Antananarivo. La région de Vakinankaratra a été choisie comme site d’étude parmi les 22 régions pour l’importance de son élevage porcin et de sa consommation de viande. En effet, deux formes d’élevage porcin sont généralement pratiquées dans la région: l’élevage de type familial où les animaux sont élevés dans une porcherie et l’élevage en semi-liberté, où les animaux sont mis en liberté mais possèdent un abri. Le Centre Hospitalier Régional de Référence d’Antsirabe (CHRR), lieu de collecte et d’analyses des prélèvements, se trouve en plein centre-ville.

### Type et durée de l’étude

Il s’agit d’une étude descriptive transversale d’une durée de 6 mois, menée entre Avril et Septembre 2013.

### Population d’étude

La population d’étude était constituée d’individus provenant de la Région de Vakinankaratra, cliniquement suspects de cysticercose, référés au laboratoire de Biologie Médicale du Centre Hospitalier Régional de Référence d’Antsirabe par leur médecin traitant respectif. L’échantillonnage des patients a été effectué selon un mode de convenance. Tous les patients, suspects cliniquement de cysticercose, venus au laboratoire pendant la période de l’étude ont été recrutés. Toutefois, les individus refusant de participer à l’étude ou présentant des crises convulsives fébriles n’ont pas été inclus.

### Données et échantillons

***Collecte des données:*** Chaque patient inclus dans l’étude a été interviewé par le personnel médical formé. Un questionnaire clinique individuel a été soigneusement rempli permettant de recueillir les données sociodémographiques et cliniques suivantes: 1) *Caractéristiques sociodémographiques et culturelles:* âge, sexe, zone d’habitation, profession, possession de jardin potager, type d’engrais utilisé, possession et utilisation de latrines, type de latrines utilisées, élevage de porc, type d’élevage (familial ou semi-liberté); 2) *Habitudes quotidiennes:* source d’eau utilisée, lavage des mains, type d’habitation, consommation de viande de porc, type de viande de porc consommée, mode de cuisson; 3) *Paramètres cliniques:* notion de céphalées chroniques sévères, notion d’épilepsie récente, notion de perte de conscience, présence de nodules sous-cutanés, cysticercose antérieure diagnostiquée.

Le choix des facteurs de risque analysés dans notre étude est basé sur les facteurs de risque connus dans la littérature.

***Collecte et traitement des échantillons:*** Tous les échantillons ont été collectés au laboratoire de Biologie Médicale du Centre Hospitalier Régional d’Antsirabe. Pour chaque sujet inclus, la procédure était: 1) *Prélèvement des échantillons sanguins:* 5ml de sang a été prélevé au pli du coude, recueilli sur tube sec; 2) *Traitement des échantillons:* centrifugation des échantillons à 3.000tr/min pendant 15 minutes à température ambiante avant de récupérer le sérum et de l’aliquoter; 3) Congélation à -20°C pour les échantillons dont l’analyse était différée

***Analyse des échantillons:*** La technique de Western blot a été réalisée sur les échantillons de sérum à l’aide du kit commercial **Cysticercosis Western Blot IgG^®^** et selon les instructions du fabricant (CYS-WB96G, 96 tests, LD Bio Diagnostic, Lyon, France). Les échantillons positifs présentent au moins 2 bandes parmi les 6 bandes suivantes: P6-8, P12, P23-26, P39, P45 et P50-55. Aucune bande n’est visible pour les échantillons négatifs.

### Analyse statistique

L’ensemble des données a été saisi sur une base informatique, créée sur Excel 2007, puis analysé avec le logiciel Epi-Info 7. Une analyse statistique descriptive a été réalisée et la séroprévalence évaluée avec un intervalle de confiance à 95%. Le test du Chi 2 avec un degré de significativité à 0,05 a été utilisé pour rechercher l’existence d’une corrélation entre la séropositivité et certains facteurs de risque potentiels.

### Ethique de l’étude

Avant le début de l’étude, plusieurs séances d’information ont été menées dans le Centre Hospitalier Régional de Référence d’Antsirabe afin de présenter et d’expliquer les objectifs de l’étude aux médecins praticiens exerçant dans la région. Un consentement éclairé et écrit a été signé par tous les participants ou par leurs tuteurs légaux pour les individus de moins de 18 ans. Tous les prélèvements ont été anonymisés par un code d´identification unique attribué à chaque personne incluse dans l´étude.

## Résultats

Durant la période d’étude, 250 patients ont effectué l’analyse au laboratoire. Parmi eux, 237 patients ont été retenus car répondant aux critères d’inclusion de l’étude, constitués de 42,2% d’hommes (n = 100) et de 57,8% de femmes (n = 137), avec un sex ratio de 0,72. Cent soixante-quatre personnes (164), soit 69,2%, provenaient de la région urbaine contre 30,8% de la région rurale. Les patients étaient âgés de 1 à 70 ans avec une moyenne de 26,1 ans. Trente-cinq (35) des 237 patients inclus ont présenté un test positif par Western blot, donnant une séroprévalence de la cysticercose de 14,8%.

Le profil sociodémographique de la population d’étude en rapport avec l’âge, la profession, l’habitation et les informations relatives à l’hygiène est exposé dans le Tableau 1. L’accès de la population étudiée à l’eau varie. Ainsi, la source principale d’eau était constituée de bornes fontaines pour 45,5% des participants (n = 108), de puisards pour 19,9% (n = 47) et seulement 18,5% (n = 44) possédaient une source d’eau potable provenant de la société de distribution d’eau. Parmi les individus inclus, seuls 14,35% (n = 34) lavaient systématiquement leur mains avec de l’eau et du savon après les selles. Le Tableau 2 nous montre la distribution de la population étudiée selon leur pratique et leurs connaissances en rapport avec le porc ainsi que les paramètres cliniques qui ont motivé leur médecin à effectuer l’analyse. Ainsi, 96,6% (n = 229) des patients consommaient de la viande de porc et parmi eux, 65,5% (n = 155) des sujets ont admis avoir mangé de la viande ladre. La séroprévalence selon le sexe, l’âge, les conditions d’hygiène, le rapport des individus avec le porc est rapporté dans le Tableau 3. Et le Tableau 4 nous montre la séroprévalence selon les paramètres cliniques de la population étudiée. Les facteurs de risque potentiels de la cysticercose humaine, analysés dans notre étude, sont présentés dans le Tableau 5. Le risque relatif rapproché (ou odds ratio OR) est supérieur à 1 pour la consommation de viande de porc, la consommation de viande ladre, l’élevage de porc, l’utilisation de fosses perdues comme latrine, la présence de céphalées chroniques, la présence d’épilepsie et la présence de nodules sous-cutanés.

**Tableau 1:** Caractéristiques sociodémographiques de la population d’étude, Antsirabe, Madagascar

Variables	Fréquence (% ) N= 237
**Age (années)**	
1-15	67 (28,27)
16-25	65 (27,42)
26-35	40 (16,90)
36-45	35 (14,76)
46-60	24 (10,12)
61-70	6 (2,53)
**Sexe**	
Masculin	100 (42,2)
Féminin	137 (57,8)
**Habitat**	
Urbain	164 (69,2)
Rural	73 (30,8)
Urbain	164 (69,2)
Rural	73 (30,8)
**Profession**	
Privé	17 (7,2)
Fonctionnaires	17 (7,2)
Artisan	30(12,65)
Fermiers et agriculteurs	32 (13,5)
Etudiants	98 (41,3)
Ménagères	16 (6,75)
Autres	27 (11,4)
**Disponibilité des latrines**	
Non	01 (0,4)
Oui	236 (99,6)
**Type de toilettes utilisées**	
Fosses perdues	150 (63)
Fosses septiques	62 (26)
En plein air	00 (00)
Pas de réponse	25 (11)
**Source d’eau utilisée**	
Borne fontaine	108(45,5)
Puits	47 (19,9)
Ruisseaux/rivières	23(9,8)
Société publique de distribution d’eau	44 (18,5)
Autres	15 (6,3)
**Lavage des mains avec l’eau et le savon après les selles**	
Oui	34 (14,35)
Non	203 (57,65)

**Tableau 2:** caractéristiques de la population d’étude selon les connaissances et pratiques autour du porc et selon les paramètres cliniques de la population d’étude, Antsirabe, Madagascar

Variables	Fréquence N= (%)
**Consommation de viande de porc (n=237)**	
Oui	229 (96,6)
Non	8 (3,4)
**Consommation de viande ladre (n=237)**	
Oui	155 (65,5)
Non	34 (14,5)
Ne sait pas	48 (20)
**Mode de préparation de la viande (n=237)**	
Cuit	163 (68,7)
Mi cuit	22(9,3)
Cuit et mi cuit	46(19,4)
Cru	6 (2,6)
**Elevage du porc (n=237)**	
Oui	94 (40)
Non	143 (60)
**Type d’élevage (n=94)**	
En liberté	16 (14)
Dans un enclos	78 (83)
**Possession de jardin potager (n=237)**	
Oui	
81 (34,2)	
Non	156 (65,8)
Oui	81 (34,2)
**Utilisation d’engrais d’origine humaine (n=81)**	
Oui	5 (6,2)
Non	76 (93,8)
**Céphalées chroniques (n=237)**	
Oui	181 (75,95)
Non	56 (24,05)
**Epilepsie (n=237)**	
Oui	81 (34,2)
Non	156 (65,8)
**Nodules sous cutanés (n=237)**	
Oui	8 (3,4)
Non	229 (96,6)

(%) = Pourcentage, N= nombre

**Tableau 3:** Séroprévalence de la cysticercose selon le sexe, l’âge, la consommation de la viande de porc, la cuisson de la viande de porc, la source d’eau utilisée, le type de latrines et l’élevage du porc parmi les participants, Antsirabe, Madagascar

Paramètres	Individus testés	Positifs n= (%)	X2	P
**Sexe**				
Masculin	100	20 (20)	1,0	
Féminin	137	15 (10,94)	3,76	0,05
**Age (en années)**				
1-15	67	9 (13,43)	1,0	
16-25	65	9 (13,84)	0,004	0,94
26-35	40	6 (15,00)	0,05	0,82
36-45	35	6 (17,14)	0,25	0,61
46-60	24	4 (16,66)	0,15	0,69
61-70	6	1 (16,66)	0,04	0,82
**Consommation de la viande de porc**				
Non	8	1 (12,5)	1,0	
Oui	229	34 (14,84)	0,03	0,85
**Consommation de viande de porc ladre**				
Non	34	6 (17,64)	1,0	
Oui	155	27 (17,41)	0,01	0,97
Ne sait pas	48	2 (4,16)	4,10	0,04^+^
**Cuit la viande avant de manger**				
Non	6	2 (33,33)	1,0	
Oui	231	28 (12,12)	2,38	0,12
**Source d’eau utilisée**				
Société publique de distribution d’eau	44	7 (15,90)	1,0	
Borne fontaine	108	13 (12,03)	0,41	0,52
Ruisseau	23	6 (26,08)	1,00	0,31
Puits	47	8 (17,02)	0,02	0,88
Autres	15	1 (6,66)	0,81	0,36
**Types de latrines**				
Fosses septiques	62	7 (11,29 )	1,0	
Fosses perdues	150	27 (17,76)	1,38	0,23
Pas de réponse	25	1(4,00)	1,13	0,28
**Elevage du porc**				
Non	143	17 (11,88)	1,0	
Oui	94	18 (19,14)	2,37	0,12

X2: Khi deux, P: valeur de p

**Tableau 4:** Séroprévalence de la cysticercose selon les paramètres cliniques, Antsirabe, Madagascar

Paramètres	Individus testés	Positifs (%)	X2	p
**Céphalées chroniques**				
Non	56	7 (12,5)	1,0	
Oui	181	28 (15,46)	0,29	0,58
**Epilepsie**				
Non	156	22 (14,10)	1,0	
Oui	81	13 (16,04)	0,16	0,68
**Nodules sous cutanés**				
Non	229	30(13,10)	1,0	
Oui	8	5 (62,5)	14,9	0,0001^+^
**Résultat de cysticercose positif par test ELISA (avant l’inclusion)**				
Non	185	19 (10,3)	1,0	
Oui	52	16 (30,76)	13,5	0,0002^+^

n= Nombre, X2: Khi deux, P= valeur de p; association significative entre les variables si p<0,05

**Tableau 5:** Comparaison de l’odds ratio de la séroprévalence selon le sexe, le type de latrines, la consommation de la viande de porc et de la viande de porc ladre, la préparation de la viande de porc, le mode de cuisson, l’élevage de porc et les paramètres cliniques de la population d’étude, Antsirabe, Madagascar

Paramètres	Odds ratio	X2	IC 95%	P
Masculin ≠ Féminin	0,49	3,76	0,23-1,01	0,05
Fosse septique ≠ Fosse perdue	1,02	0,003	0,46-2,24	0,95
Fosse septique ≠ autres types de latrines	0,23	2,31	0,02-1,79	0,12
Mange de la viande de porc ≠ ne mange pas	1,22	0,03	0,14-10,23	0,85
Mange de la viande ladre ≠ ne mange pas	1,21	0,19	0,51-2,86	0,65
Mode de cuisson cuit ≠ mode de cuisson mi-cuit	0,59	1,83	0,28-1,26	0,17
Elevage de porc ≠ non élevage	1,79	2,55	0,87-3,68	0,10
Présence de céphalées chroniques ≠ absence	1,28	0,29	0,56-3,11	0,58
Présence de nodules≠ absence	11,05	14,98	2,51-48,66	0,0001^+^
Présence d’épilepsie ≠ absence	1,16	0,16	0,55-2,45	0,68

X2: Khi deux, P: valeur de p, IC 95%: intervalle de confiance à 95%

## Discussion

Cette étude a permis de déterminer la séroprévalence et les facteurs de risque potentiels de la cysticercose dans une population constituée de sujets porteurs de signes cliniques évocateurs issue de la région de Vakinankaratra. Elle montre que la cysticercose est encore une maladie très présente à Madagascar. La séroprévalence déterminée à l’aide de la technique par Western blot est de 14,8%. Ce chiffre est presque similaire à celui rapporté par Andriatsimahavandy et al. en 2003 et est en accord avec leurs résultats qui montrent que la prévalence de la cysticercose est plus élevée dans les régions des Hautes-Terres de Madagascar [13]. Ce résultat est proche des 14,8% obtenus par Bucardo et al. au Mexique [14] et des 16% obtenus par Prabhakaran et al. en Inde [15], qui ont tous deux utilisé le Western blot. D’autres études utilisant un test ELISA ont permis d’obtenir des valeurs semblables, soit 9,6% au Nigéria [16] et 16,5% dans la communauté de Mbulu en Tanzanie [17]. Par contre la séroprévalence trouvée dans notre étude est plus élevée que celle trouvée dans d’autres études, menées dans certains pays endémiques de l’Afrique de l’ouest, comme au Togo (2,3%), au Bénin (1,4%) ou encore au Cameroun (entre 0,7% à 2,4%) [18]. Dans les pays d’Afrique de l’Est, par contre, le taux de prévalence de la cysticercose est très élevé atteignant jusqu’à 30% au Burundi, 45,3% dans le district de Mbozi en Tanzanie [19] ou encore 20,3% au Mozambique [20] mais avec un test ELISA Anticorps comme technique d’analyse. Dans certains pays d’Asie, la prévalence de la cysticercose est très variable, allant de 1,65 à 3,97% en Indonésie atteignant même 16,4% en Chine [15]. Les résultats des études séro-épidémiologiques peuvent être influencés par plusieurs facteurs, notamment, le choix des outils diagnostiques, leur sensibilité, leur spécificité et la facilité d’utilisation du produit lors des analyses au laboratoire. En comparant les différentes études, les techniques de détection des anticorps donnent une prévalence plus élevée par rapport aux techniques de détection des antigènes [20]. Toutefois, elles sont toujours considérées comme des outils de dépistage appropriés dans la population puisqu’elles indiquent l’exposition à une maladie.

La séroprévalence de notre étude est également en accord avec la littérature qui démontre que dans les villages les plus endémiques, plus de 10% de la population est séropositive et que cette séropositivité peut même atteindre 25% [16,21]. Ces résultats confortent encore la position de Madagascar comme un pays endémique de la cysticercose, pathologie tropicale posant un problème majeur de santé publique. La prévalence cysticerquienne semble liée à l’intensité de l’élevage [13]. La région de Vakinankaratra située sur les Hautes-Terres centrales présente des structures socioéconomiques et écologiques propices aux activités propres de la population agro-pastorale. Comme en témoigne nos résultats, 40% de la population étudiée pratiquent l’élevage. La riziculture est associée à un important élevage de bovins et de porcins parfois en liberté comme en témoignent les 14% d’élevage en liberté retrouvés dans notre étude. Ces résultats indiquent également que les efforts déjà menés par le Ministère de la Santé doivent être renforcés. L’intensification des moyens de prévention de la maladie est primordiale. La promotion de comportements favorables à la santé par la production de supports d’informations et des séances de sensibilisation sont également nécessaires en insistant sur les modes de contamination et de transmission de *T solium*; les différents moyens de prévention; la promotion de la construction et de l’utilisation de latrines et la conception d’un guide à l’usage scolaire.

Dans cette étude, une séroprévalence élevée des anticorps anti-cysticerques chez les hommes a été retrouvée, évaluée à 15,46% soit 20 positifs sur 100 testés. Sans différence significative selon le sexe (p > 0,05), les deux sexes peuvent être infectés, le sexe n’étant pas un facteur de risque. Ces résultats peuvent suggérer que dans cette région de Madagascar, l’homme et la femme pratiquent les mêmes activités. Alors que généralement [13,16], ce sont les femmes qui présentent un risque élevé de contracter la maladie, en lien avec leurs activités telles que le nettoyage domestique, la préparation des aliments ou encore les travaux de jardinage. Ces résultats diffèrent des données observées en 2003 à Madagascar [13], de celles de 2013 au Nigéria [16], de celles de Carrique-Mas et al. obtenues en 2001 en Bolivie [22] où une prévalence féminine de la maladie a été observée.

Concernant la séroprévalence de la cysticercose par rapport aux différents groupes d’âge, elle est de 17,14% dans la tranche d’âge de 36 à 45 ans. La tranche des 46 à 60 ans montre elle aussi une prévalence élevée (16,66%). En Tanzanie, Mwanjali et al. a également rapporté un pic élevé de la prévalence cysticerquienne dans la tranche d’âge de 36 à 60 ans [19]. Une étude récente a indiqué des taux élevés d’infection active chez les personnes âgées qui pouvaient s’expliquer par une faible réponse immunitaire [23]. De plus, selon Moro et al. [24], la séroprévalence de la maladie augmente à mesure que l’âge augmente. La plus faible séroprévalence est observée dans la tranche des moins de 15 ans (13,43%). Toutefois, cette différence n’est pas significative, (p > 0,05), indiquant que dans les mêmes conditions et à tout âge, on peut contracter la maladie. Des études antérieures, menées en milieu hospitalier dans la capitale, avaient déjà montré que la cysticercose peut toucher des enfants âgés de 2 ans. Les résultats de ces différentes études menées à Madagascar montrent que l’affection est fréquente chez les moins de 10 ans avec des prévalences de l’ordre de 5 à 10% [13,25,26].

D’après nos résultats, la séroprévalence est élevée chez les individus qui utilisent les fosses perdues comme latrine (11,76%) ainsi que chez les personnes qui utilisent l’eau du puits (17,02%) ou l’eau des rivières (26,08%) comme source d’eau. Cependant, elle est également élevée chez ceux qui ne consomment pas de viande de porc ladre ou n’ont pas reconnu en avoir mangé (Tableau 3). Ces données concordent avec la littérature qui affirme que l’absence d’infrastructures sanitaires adéquates joue un rôle important dans la pérennisation du cycle de transmission de *T. solium* [16]. La consommation de viande de porc est importante dans la transmission des infections à *T. solium*. Dans notre étude, la séroprévalence de la cysticercose est plus élevée chez les consommateurs de viande de porc (14,84%) par rapport à ceux qui n’ont mangent pas (12,5%), mais cette différence est non significative (Tableau 5). Ces résultats peuvent s’expliquer par le fait que la plupart des sujets qui ont participé à l’étude ont bien cuit leur viande avant de la consommer. En effet, cette pratique détruit complètement les cysticerques et permet de prévenir la transmission de la pathologie. La séroprévalence chez les individus qui ont présenté des céphalées chroniques est élevée (15,46%) par rapport à ceux qui étaient non céphalalgiques. Les céphalées sont des symptômes fréquents mais non spécifiques de la cysticercose et sont généralement associées aux épilepsies. Les épilepsies, secondairement généralisées ou d’emblée généralisées sont les manifestations les plus fréquentes de la cysticercose. Elles sont dues, dans la plupart des cas à des kystes dégénératifs ou à des granulomes calcifiés [27]. Une étude réalisée à Antananarivo entre 1993 et 1995 a montré que 20 à 25% des épilepsies inaugurales de l’adulte à Madagascar étaient en relation avec la cysticercose [28].

Dans notre étude, la prévalence de la cysticercose est de 16,04% chez les individus qui ont eu un épisode d’épilepsie contre 14,10% chez ceux qui n’en ont pas eu (Tableau 4 et Tableau 5). Ces données sont faibles par rapport à ceux de Mwanjali (Sud de la Tanzanie), où une prévalence de 22,8% a été observée chez les sujets présentant une épilepsie [17]; 37,5% en Inde de l’ouest (par ELISA) [29]; 44,6% au Cameroun [18], 22,5% au Pérou (par EITB) [30] et 31,3% au Mexique (par EITB) [31]. Au contraire, elles sont similaires aux 14% retrouvés au Nord de la Tanzanie [32]. La différence retrouvée dans ces résultats peut résulter de l’influence de la méthode de définition de l’épilepsie utilisée pour chaque étude. L’existence d’une association entre les symptômes cliniques et la positivité du test a été recherchée chez les participants présentant des nodules sous cutanées, et semble significative avec un taux de 62,5%. Cette association est aussi significative pour les personnes qui ont eu un résultat positif par un test ELISA Anticorps six mois avant l’analyse (30,76%) (p < 0,05) (Tableau 4). Selon Praet et al. le pourcentage de séro-réversion des anticorps dépend du nombre d’exposition de chaque individu au parasite, mais aussi de leur âge et de leur statut immunologique. La séro-négativation des anticorps est de 60% à la première exposition, diminuant à 20% à la deuxième exposition et ainsi de suite [23]. Nos résultats peuvent donc suggérer l’existence de ré-infestations multiples chez les sujets de notre étude. Notre étude présente toutefois quelques limites comme le mode de recrutement de nos patients et le questionnaire utilisé qui peut donner dans certains cas des fausses réponses positives ou négatives.

## Conclusion

Les résultats de notre étude confirment que la cysticercose est encore très présente à Madagascar. L’élevage du porc et la consommation de sa viande demeurent important dans cette partie de l’île. La séroprévalence de 14,8% retrouvée dans notre étude est élevée. Elle est significativement élevée chez les sujets qui ont présenté des nodules sous-cutanés et chez ceux qui ont eu un résultat antérieur positif par test ELISA. Vu l’immensité du pays et la grande diversité des régions, l’étude mérite d’être étendue à une plus grande échelle permettant d’avoir la situation séro-épidémiologique générale de la cysticercose. La détection des anticorps circulants anti-*T. solium* par Western blot est un excellent outil pour le diagnostic de la cysticercose et de la neurocysticercose, surtout dans les zones endémiques où les techniques d’imagerie comme le scanner ou l’IRM ne sont pas disponibles. L’utilisation appropriée des ces outils, associée aux renforcements des mesures de contrôle et de prévention déjà implantés seront la garantie de la lutte contre la maladie à Madagascar.

### Etat des connaissances actuelles sur le sujet

- Les derniers chiffres concernant la prévalence de la cysticercose à Madagascar datent de 2003, l’évaluant entre 7% à 20% à l’échelle nationale;
- Depuis une dizaine d’année, aucune donnée sur l’importance réelle de la cysticercose humaine et sa prévalence n’est disponible dans la grande île;
- Selon plusieurs études menées en Afrique, *Taenia solium* est probablement répandu dans la plupart des pays africains où les porcs sont élevés en liberté et où la viande de porc est consommée.

### Contribution de notre étude à la connaissance

- Mise à jour des données épidémiologiques de la cysticercose humaine dans la région de Vakinankaratra, importante par son élevage porcin et sa consommation de viande de porc;
- La cysticercose est encore très présente à Madagascar, même si l’étude a été effectuée sur une population ciblée;
- Etude utilisant la technique Western blot pour déterminer la séroprévalence de la cysticercose chez les patients cliniquement suspects de la maladie.

## Conflits d’intérêts

Les auteurs ne déclarent aucun conflit d’intérêts.

## Remerciements

Nous tenons à remercier vivement tous les patients qui ont bien voulu participer à l’étude ainsi que tout le personnel du laboratoire d’analyses médicales du Centre Hospitalier Régional de Référence d’Antsirabe pour leur coopération et leur assistance. Nos sincères remerciements vont aussi à l’endroit de tous les médecins qui ont bien voulu référé leurs patients à l’hôpital. Nous tenons également à remercier la société LDBIO Diagnostics pour les kits d’analyse. Nous remercions la Fondation Mérieux d’Antananarivo et le Centre d’Infectiologie Charles Mérieux de Madagascar pour leur support financier et technique.
